# Minimally-invasive resection of deep-seated subcortical and intraventricular lesions using an integrated camera-port system: A retrospective case series

**DOI:** 10.1371/journal.pone.0348673

**Published:** 2026-05-11

**Authors:** Robert G. Briggs, Danielle Levy, Ryan S. Chung, David J. Cote, Ishan Shah, David Gomez, Kevin Liu, James Sobieski, Jonathan Sisti, Gabriel Zada

**Affiliations:** 1 Department of Neurological Surgery, Keck School of Medicine of the University of Southern California, Los Angeles, California, United States of America; 2 Keck School of Medicine of USC, University of Southern California, Los Angeles, California, United States of America; Queen#39;s University Belfast, UNITED KINGDOM OF GREAT BRITAIN AND NORTHERN IRELAND

## Abstract

Minimally invasive neurosurgery using port-based systems has gained popularity over the past decade to access deep-seated lesions within the brain. These devices typically necessitate use of separate optical and illumination systems. An exception is the Aurora Surgiscope (Integra Corp.) which is a single-use, camera-port retractor with integrated lighting and visualization capabilities. We present our retrospective single-center series using this technology for the treatment of a diverse set of intra-axial lesions to demonstrate the safety and effectiveness of the device for removing deep-seated intracranial targets. A retrospective review was conducted for all adult patients who underwent removal of an intra-axial lesion using the Aurora Surgiscope camera-port from 01/11/2021–31/12/2024 at our institution. Relevant demographic, radiographic, and clinical data were collected and summarized. 25 patients were included in the series (12 females, mean age 52.4 ± 17.7 years). The camera-port system was used to treat a diverse set of pathologies, including colloid cysts (n = 6/25, 24.0%), intraparenchymal hematomas (IPH) (n = 5/25, 20.0%), brain metastases (n = 4/25, 16.0%) and ependymomas/gliomas (n = 4/25, 16.0%). Mean lesion depth was 3.5 cm. Mean lesion diameter was 3.5 cm. Median surgical time was 2.4 hours. Gross total resection was achieved in 12/17 (70.6%) operations where complete resection of the tumor or colloid cyst was the surgical objective. Four patients experienced postoperative neurologic complications, including one patient with transient short-term memory loss, two patients with transient aphasia, and one patient with persistent left-sided weakness. Median post-operative length of stay was 4 days. This case series demonstrates the feasibility of using an integrated camera-port system for the treatment of deep-seated brain lesions. The technology integrates visualization and illumination capabilities with reasonable surgical outcomes.

## Introduction

Approaches to deep intra-axial and intraventricular lesions present unique operative challenges given the elevated risk of iatrogenic neurologic injury compared to more superficial lesions [1,2]. Proximity to eloquent structures, limited visualization when accessing deep or dark surgical corridors, and potential injury from prolonged retraction of surrounding healthy brain tissue are some of the challenges neurosurgeons encounter when accessing these lesions [3–6]. Deep-seated lesions which require extensive retraction often include colloid cysts, subcortical cavernous malformations, and primary and metastatic brain tumors. There remains a clear need to explore surgical technology and techniques to access these lesions while minimizing collateral damage to adjacent, healthy brain tissue.

Minimally invasive approaches have been more widely adopted over the past decade with the introduction of port-based tubular retractors as an alternative to open craniotomies [7]. Tubular retractors present a promising alternative to traditional blade retractors to establish surgical corridors to reach deep-seated lesions. Proposed advantages of these systems include their use with smaller craniotomies and dural openings, the circumferential dilation and displacement (rather than transection) of healthy brain parenchyma during insertion of the device, trans-sulcal access, and the reduced likelihood of retraction injury from the radial distribution of forces applied by the tube [8].

There are a variety of commercial tubular retractor systems, including the BrainPath system (NICO Corp, Indianapolis, Indiana) and the Viewsite Brain Access System (VBAS; Vycor Medical Inc, Boca Raton, Florida), which have been successfully adapted to treat deep-seated lesions [7,9]. Although several options exist for port-based access to subcortical lesions, the disadvantage of existing systems is that they typically require separate external lighting and visualization devices, such as a microscope, exoscope or endoscope to visualize deep targets via the collinear access provided by the port. Because of this, a light source and optical system must be maintained in a coaxial position to the tubular retractor for proper use. This has the potential to impede surgeon workflow efficiency and ergonomics, as frequent repositioning of the retractor with the light and camera sources is necessary. In addition, with endoscopes or exoscopes, bimanual techniques can be limited, as one hand is used to manipulate the camera [10].

The Aurora Surgiscope (Integra Corp, Princeton, New Jersey) is a novel, FDA-approved, single-use, port-based, navigable retractor system with an integrated camera and light source that provides exoscopic two-dimensional visualization aimed to address some of the limitations of existing commercial products. In this study, we sought to provide one of the first case series reports pertaining to the technical feasibility and effectiveness of this camera-port device for surgical access to a variety of subcortical and intraventricular lesions.

## Materials and methods

### Data collection

All adult patients (age > 18 years) undergoing surgery with documented use of the Aurora Surgiscope between 01/11/2021–31/12/2024 for an intracranial lesion at our institution were included in this study. Institutional Review Board approval from the University of Southern California was obtained for the study which adhered to all relevant ethical guidelines (IRB HS-18–00835). Patient consent was obtained for the inclusion of all relevant materials in this study. Electronic medical records were retrospectively reviewed to record pertinent clinical, radiologic, and surgical data for each patient. Data were retrospectively collected and analyzed from patients charts from 25/06/2025–31/12/2025. All data are in the manuscript and/or supporting information files

### Surgical planning

The Aurora Surgiscope system consists of three components: (1) the obturator which inserts into the port of the tubular retractor with a protruding blunt tip that can be fix to the tube and co-registered with a neuronavigation system, (2) the tubular retractor with the attached light source and camera mounted on a compact ring apparatus, and (3) a video box that projects a live camera view to an external video monitor. The camera-port system has an outer diameter of 15 mm and port lengths of either 6 or 8 cm.

Prior to surgery, patients underwent complete clinical, laboratory, and radiological evaluation. MRI or CT imaging was used in conjunction with stealth neuronavigation. Preoperative imaging was integrated into the camera-port system to allow us to plan a trajectory to the lesion of interest. For patients with an intraparenchymal hematoma (IPH), CT stealth scans were preferred for use with navigation due to their increased sensitivity for hemorrhage. For IPH cases, the planned navigation trajectory included an end-target set in the center of the IPH. For tumors, the port target was planned to the surface of the tumor. For intraventricular lesions such as colloid cysts, the port trajectory terminated in the frontal horn of the lateral ventricle at the level of the Foramen of Monro.

### Operative technique

In the operating room, patients were positioned according to the location of their lesion and the trajectory using the camera-port system. After induction for general anesthesia, patients were secured with a three-pin head clamp. We then performed the anatomic registration necessary for optical neuronavigation. When indicated, somatosensory (SSEP) and motor evoked potential (MEP) neuromonitoring were used to assess for any changes in neurologic signals during surgery. Medical diuresis was not pursued in these cases in order to maintain normal brain turgor for the port insertion. A 3–4 cm craniotomy was performed over the preplanned trajectory. The dura was opened in a cruciate fashion with an opening diameter slightly larger than the outer diameter of the Surgiscope (15 mm). Trans-sulcal dissection was performed with microscissors and bipolar forceps to facilitate insertion of the obturator and tubular retractor at the depth of sulcus after resetting the trajectory entry point. The device was then inserted in conjunction with a neuronavigation probe to monitor our depth to the target location along our preplanned trajectory through the white matter parenchyma.

After inserting the device to the target depth, the tubular retractor was secured in place using a Fukushima snake retractor arm. The obturator was then removed, providing two-dimensional visualization of the operative field through the camera port. In IPH cases, the main technical difference was that the camera-port was inserted along into the depth of the hematoma before removing the obturator. At this stage, microsurgical instruments were introduced into the port with the lesionectomy performed using a standard two-handed surgical technique in an open-air medium.

For IPH cases, the clotted blood from the hemorrhage typically expressed itself into the port, and evacuation of the hemorrhage proceeded with copious suction and irrigation until hemostasis was achieved locally. The port was gradually removed along the trajectory plane by loosening the Fukushima retractor and pulling the port back slowly. This was done in order to achieve complete IPH evacuation starting at the depth of the hematoma and working superficially. In colloid cyst cases, the cyst capsule was fenestrated and drained prior to sharp dissection of the deflated cyst wall. For solid tumors, the lesion was typically debulked in a piecemeal fashion with adjustments to visualize the operative field made by toggling the port or adjusting its depth to visualize more tumor. After completing the lesionectomy, the camera-port was toggled circumferentially and slowly withdrawn along the surgical tract to visualize the surgical cavity and evaluate for any sites of active bleeding in order to achieve hemostasis before withdrawing the device completely. At this point, the surgical site was irrigated and closed in standard multilayer fashion without the need for a postoperative drain.

Over the course of this series, we made several adjustments to our surgical strategy. First, we found that docking the port at the depth of an IPH (as opposed to the hematoma surface) proved more effective at disrupting the clot internally. This made evacuating hematomas faster and easier. We generally avoided this strategy for tumors or cysts, though, in part because docking the port at the surface of the tumor or cyst capsule allowed us to perform an extracapsular dissection for gross removal of the lesion if small enough. If the lesion was sizeable, we would still plan to dock the port on the capsule surface of the lesion in order to open the lesion capsule for internal debulking. We found that toggling the port could be done easily in different directions to achieve a maximally safe resection with intermittent reference to the neuronavigation or blue light endoscope (when indicated) to confirm gross resection. Finally, we found it was relatively easy to adapt to the 2D exoscopic view provided by the port. We attribute this to our team’s extensive background in endoscopic endonasal skull base work for which 2D endoscopic views are the norm. Neurosurgeons without this background may require some time adjusting to this viewpoint given the lack of 3D microscopic detail.

### Statistical analysis

Categorical data were summarized as counts/percentages, and continuous data were reported as medians/ranges. Extent of resection was defined as gross total resection (GTR) or subtotal resection (STR) as determined by a board-certified neuroradiologist who reviewed all pre- and post-operative MRI or CT scans.

## Results

### Patient characteristics

**Fig 1:**
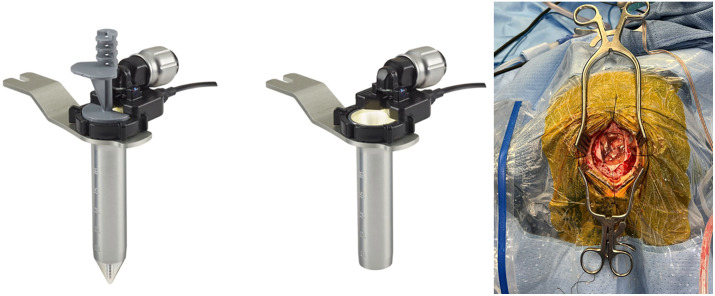
Images of the Aurora Surgiscope system with and without the navigable obturator inserted into the surgical port. The craniotomy insert demonstrates the 4-cm mini-craniotomy needed to accommodate the camera-port system.

Twenty-five patients underwent either resection or biopsy of intracranial lesions using the Aurora Surgiscope camera-port system (Fig 1) between November 2021 and December 2024. This included 13 male patients and 12 female patients. The average age of patients was 52.4 ± 17.7 years (Range: 20–83 years). Patients with a variety of lesions, including colloid cysts (n = 6/25, 24.0%), hematomas (n = 5/25, 20.0%), brain metastases (n = 4/25, 16.0%), and subcortical ependymoma/gliomas (n = 4/25, 16.0%), were treated surgically using this technology. Mean lesion depth below the cortical surface was 3.53 cm. Mean lesion diameter was 3.46 cm. 5-alminolevulinic acid (ALA) was administered pre-operatively for four suspected glioma cases. During these surgeries, a blue-light endoscope was inserted through the camera-port system to guide further tumor resection. This required us to first turn off the white-light illumination source from the Aurora Surgiscope to identify residual tumor fluorescence revealed by the blue-light endoscope. If fluorescence was observed with blue-light endoscopy, then we would resume the tumor resection by toggling back to the white light provided by the Surgiscope port. Toggling of the white light source was performed by the circulating nurse as the on/off switch for the light source is not located on the port device itself. Overall, there were no notable disruptions to the surgical workflow or ergonomics of these operations when toggling between light sources. Table 1 summaries patient demographic, clinical, and lesion data.

**Table 1:** Patient demographic, clinical, radiologic, and surgical characteristics.

Age (years)	Sex	Presentation	Pathology	Location	Lesion Size	Lesion Depth	Approach	5-ALA	Port Length	Surgical Time	Resection	Discharge (POD)	Complications
60	M	Seizure	Metastasis from lung	R Frontal	2.7 cm	3.9 cm	R frontal craniotomy for tumor	N	NA	2:32	GTR	5	None
73	F	R sided weakness	Metastasis from Lung	L Parietal	3.3 cm	2.3 cm	L parietal craniotomy for tumor	N	8 cm	2:49	STR	2	None
55	M	Growing mass	Metastasis from Renal	L Parietal	4.4 cm	3.1 cm	L parietal craniotomy for resection	N	6 cm	3:19	GTR	3	None
67	F	L sided weakness	Metastasis from Bladder	R Parietal	2.6 cm	1.7 cm	R parietal craniotomy for port resection x 2	N	6 cm	1:49	GTR	11	None
42	M	HA, Seizure	GBM	Multifocal, R Corpus Callosum	1.8 cm, 3.1 cm	4.8 cm	R parietal craniotomy	Y	8 cm	3:40	STR	2	None
67	M	R sided weakness	GBM	Multifocal, L Parietal	3.8 cm, 3.9 cm	2.6 cm	L parieto-occipital craniotomy	Y	6 cm	1:34	Biopsy	3	None
71	M	Memory Issues	GBM	L Temporal	3.9 cm	3.8 cm	L parieto-occipital craniotomy	Y	8 cm	3:28	STR	4	None
39	M	R sided weakness	Astrocytoma	L Thalamic and Midbrain	2.3 cm	6.1 cm	L frontal craniotomy for cyst fenestration and biopsy	N	NA	2:38	Biopsy	6	Transient aphasia
27	F	Recurrence, AMS	Ependymoma	L Parietal	6.6 cm	2.3 cm	L parieto-occipital craniotomy	Y	6 cm	4:13	STR	3	None
37	M	HA	Neurocytoma	Central	5.0 cm	5.2 cm	R frontal craniotomy, port approach	N	NA	5:29	GTR	22	Left sided weakness
61	F	Vision Loss	Choroid Plexus Papilloma	R Parietal	2.0 cm	0.5 cm	R parietal craniotomy	N	8 cm	2:46	STR	2	None
53	M	HA, R sided weakness, Seizures	Colloid Cyst	Third Ventricle	1.7 cm	5.5 cm	R frontal craniotomy for colloid cyst	N	8 cm	5:37	GTR	4	None
20	F	HA	Colloid Cyst	Third Ventricle	1.2 cm	4.4 cm	R frontal craniotomy for colloid cyst	N	6 cm	2:24	GTR	8	Short term memory difficulty, cognitive deficits, hydrocephalus
25	F	HA, hydrocephalus	Colloid Cyst	Third Ventricle	.7 cm	6.5 cm	R frontal craniotomy for colloid cyst	N	6 cm	2:20	GTR	3	None
71	F	HA	Colloid Cyst	Third Ventricle	1.8 cm	4.9 cm	L frontal craniotomy	N	NA	1:35	GTR	4	Transient aphasia
40	M	HA	Colloid Cyst	Third Ventricle	0.9 cm	6.2 cm	R frontal craniotomy for colloid cyst	N	NA	1:56	GTR	2	None
62	M	HA, ataxia	Colloid Cyst	Third Ventricle	1 cm	7.2 cm	R frontal craniotomy	N	6 cm	2:11	GTR	4	None
41	M	L sided weakness, AMS	IPH	R Frontal	7.3 cm	1.1 cm	R frontal craniotomy	N	NA	2:01	GTE	10	None
64	M	L sided weakness	IPH	R Basal Ganglia	7.4 cm	2.5 cm	R frontal craniotomy	N	NA	1:50	GTE	16	None
41	F	R sided weakness, aphasia, coma	IPH	L Basal Ganglia	6.2 cm	3.3 cm	L frontal craniotomy for ICH	N	8 cm	1:57	GTE	30	None
75	F	HA	IPH	R Temporal	5.7 cm	1.2 cm	R temporoparietal craniotomy	N	6 cm	1:01	GTE	5	None
83	M	AMS	IPH	R Parietal	4.1 cm	1.0 cm	R temporoparietal craniotomy for ICH	N	NA	2:00	GTE	24	None
35	F	Seizure	Cavernous Malformation	R Frontal	2.5 cm	3.5 cm	R supraorbital craniotomy	N	NA	3:41	GTR	2	None
33	F	AMS	Cavernous Malformation	Lateral Ventricle	4.9 cm	3.1 cm	L parieto-occipital craniotomy	N	8 cm	3:36	GTR	4	None
64	F	AMS	Gliosis	L Temporal	2.5 cm	1.6 cm	L temporal craniotomy for biopsy	N	6 cm	1:32	Biopsy	10	None

*Abbreviations: M = male; F = female; GBM = glioblastoma; IPH = intraparenchymal hematoma; R = right; L = left; N = no; Y = yes; NA = not applicable; GTR = gross total resection; GTE = gross total evacuation*

### Surgical and clinical outcomes

For IPH cases (5/25), complete evacuation was achieved in all five patients (100%). Similarly, GTR was achieved in 12/17 operations where complete resection of the tumor or colloid cyst was the surgical objective (70.6%). Diagnostic biopsy alone was performed in three glioma patients (3/25, 12.0%). In each of the 5-ALA cases, 5-ALA aided in the visualization and resection of additional tumor using an angled blue light endoscope. No patients experienced intraoperative complications. Intraoperative neuromonitoring was stable in all cases. No cases required conversion to traditional open approaches.

There were four instances of post-operative surgical complications (4/25, 16.0%) and three instances of non-surgical complications (3/25, 12.0%). Post-operative surgical complications included two patients experiencing transient short-term memory loss and cognitive deficits following resection of a colloid cyst; one patient developing persistent left-sided weakness following resection of a neurocytoma approached via a right frontal craniotomy; and one patient experiencing transient aphasia following resection of a left thalamic astrocytoma approached via a left frontal craniotomy. Non-surgical complications included pneumonia in two patients and a urinary tract infection in one patient. Median surgical time was 2.4 hours. Median post-operative length of stay was four days. All patients reported improvement in their presurgical symptoms by their first post-operative clinic visit. Patient outcome data is also summarized in Table 1**.**

### Illustrative cases

#### Case 1 – intraventricular colloid cyst.

**Fig 2:**
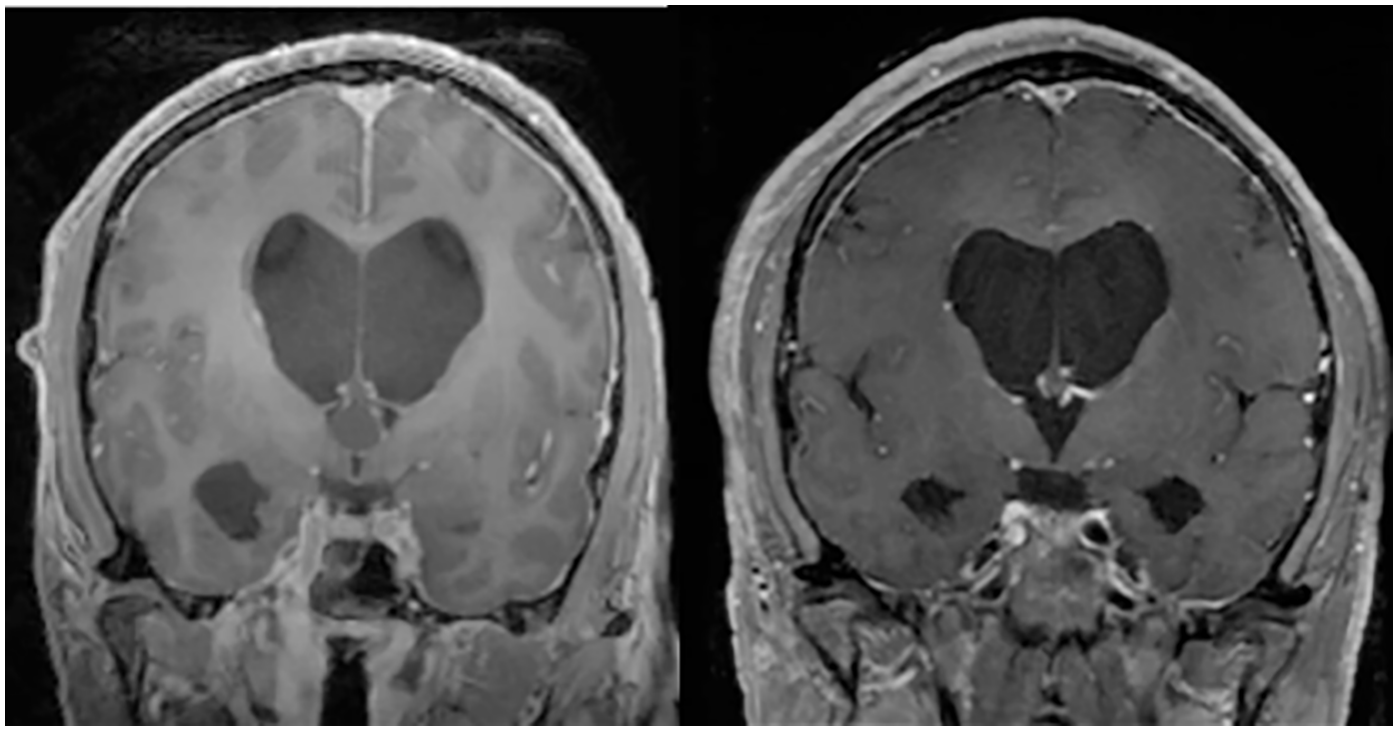
Examples of pre- and post-operative images after Aurora Surgiscope-based treatment of various intracranial pathologies. Coronal T1 post-contrast magnetic resonance images demonstrating complete resection of a third ventricular colloid cyst.

A 62-year-old male presented with headaches and memory loss. Initial imaging revealed a suspected third ventricular colloid cyst with ventriculomegaly. Given that the colloid cyst was eccentric to the right side of the third ventricle, a right frontal craniotomy was selected for a trans-sulcal, port-based resection (15-mm diameter, 8-cm port length). The lesion capsule depth from the brain surface measured 6.2 cm. The patient’s hospital course was uncomplicated, and he was discharged home on post-operative day (POD) three. At his initial follow-up visit in clinic, he reported complete resolution of his headaches and memory issues. The patient did not require further surgery for CSF diversion. This case is illustrated in Video 1. Pre-operative and post-operative coronal T1 post-contrast MRI images demonstrating GTR of the colloid cyst are shown in Fig 2.

**Video 1.** Aurora Surgiscope-based resection of a third ventricular colloid cyst in a 62-year-old man presenting with headaches and memory loss.

#### Case 2 – subcortical IPH.

**Fig 3:**
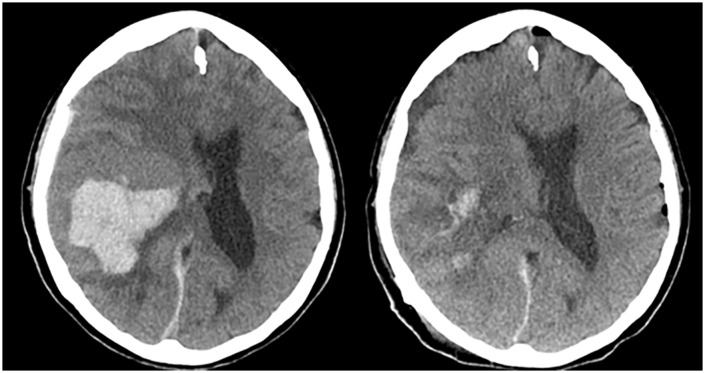
Examples of pre- and post-operative images after Aurora Surgiscope-based treatment of various intracranial pathologies. Non-contrast axial CT scans demonstrating evacuation of an intraparenchymal hematoma.

A 75-year-old female with a history of hypertension and diabetes presented after acute-onset of headache and lethargy. Initial Glasgow Coma Scale (GCS) on examination was 10 (E1M6V3). CT imaging demonstrated a large right posterior temporal IPH. CT angiography was negative for any active vascular lesion. The patient was taken for surgical evacuation of the IPH using the Aurora Surgiscope System (15-mm diameter, 6-cm port length). The patient underwent a right temporoparietal craniotomy with the hematoma 1.2 cm from the depth of the cortical surface. Post-operative CT demonstrated successful evacuation of the IPH with minimal residual blood products within the hematoma cavity. Her post-operative GCS improved to 13 (E3M6V4). She was discharged to a nursing facility on POD5. Pre-operative and post-operative non-contrast axial CT images showing evacuation of the IPH are shown in Fig 3.

#### Case 3 – subcortical high-grade glioma.

**Fig 4:**
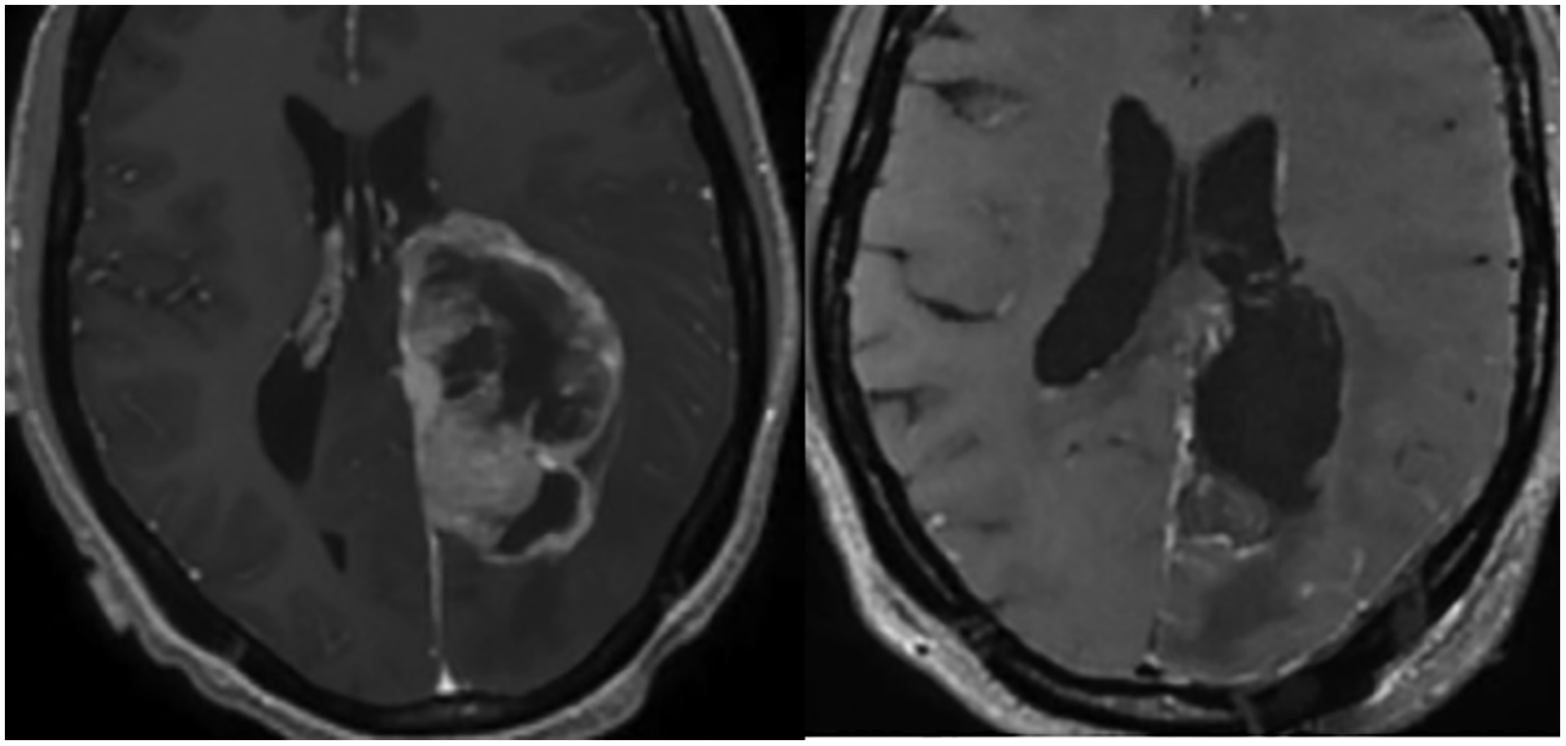
Examples of pre- and post-operative images after Aurora Surgiscope-based treatment of various intracranial pathologies. Axial T1 post-contrast magnetic resonance images showing the results after resection of a left parieto-occipital glial neoplasm.

A 27-year-old female with a history of a brain tumor presented with headaches, nausea, vomiting, and encephalopathy. She was found on MRI to have an enhancing lesion in the left parieto-occipital lobe concerning for a high-grade glioma. She underwent a left parietal craniotomy for port-based resection of the lesion using the Aurora Surgiscope (15-mm diameter, 8-cm port length). 5-ALA was administered preoperatively and blue-light endoscopy was used to ensure maximal safe resection of the tumor. Neuromonitoring remained stable throughout the operation. Post-operative imaging demonstrated STR with <10% residual of the mass. Pathology revealed a high-grade ependymoma. The patient’s post-operative course was uncomplicated, and she was discharged to home on POD3. This case is illustrated in Video 2. Pre-operative and post-operative axial T1 post-contrast MR images demonstrate the results of the port-based resection of the glial neoplasm in Fig 4.

**Video 2.** Aurora Surgiscope-based resection of a left parieto-occipital high-grade glioma in a 27-year-old woman presenting with acute encephalopathy.

## Discussion

In this study, we demonstrate the technical feasibility and efficacy of the Aurora Surgiscope camera-port system for resection of a variety of deep intracranial lesions. In all 25 cases, the lesions were adequately visualized and resected using this device without requiring conversion to another approach or reliance on additional visualization, with the exception of the blue-light endoscope to aid in additional resection of deep-seated subcortical gliomas. Our findings are consistent with the literature demonstrating the technical feasibility of the Aurora Surgiscope system in treating deep intracranial lesions while minimizing perioperative complications associated with more traditional open cranial approaches [11–13]. Furthermore, our study demonstrates similar extent of resection and complication rates to other tubular retractor systems, such as the BrainPath, VBAS, and METRx (Table 2) [9,14].

**Table 2:** Comparison of features for common port-based tubular retraction systems.

Retractor System	Dimensions (Diameter & Length)	Applications	Key Features	Pros	Cons
BrainPath® (NICO)	11 mm, 13.5 mm (5 cm, 6 cm, 7.5 cm,9.5 cm lengths)	Tumors, IPH, biopsy, deep-seated lesions	Blunt-tip, atraumatic dissection, neuronavigation-compatible	Minimizes tissue damage, well-studied, widely used	Requires microscope or exoscope for visualization
Vycor VBAS™ (Vycor Medical)	6mm, 12 mm, 17 mm, 21 mm, 28 mm (3,5,7 cm length)	Tumors, hematomas, biopsies	Transparent walls, compatible with microscopes & endoscopes	Direct visualization through walls, multiple sizes	Requires external visualization, cortical strain possible
METRx® (Medtronic)	14 mm, 18 mm, 22 mm (various lengths)	Spine surgery, deep brain access	Sequential dilation, minimally invasive	Reduces muscle & tissue damage, commonly used in spine surgery	Limited deep brain use, requires dilators
Aurora Surgiscope™ (Integra Corp.)	15mm x 60 mm 15mm x 80 mm	Tumors, hematomas, biopsy	Built-in endoscope, retraction, real-time visualization	All-in-one system, minimizes need for extra tools, enhances precision	Newer system, less clinical data compared to BrainPath

Subcortical and intraventricular lesions can be challenging to treat given their ability to infiltrate eloquent brain tissue, arise in areas with limited cisternal access, and increase the risk of neurological deficits if not carefully resected [2]. Traditionally, a neurosurgeon would perform blunt white matter separation with a corticotomy, place flat metal retractors within the brain to access the lesion of interest, and dissect towards the lesion using bipolar forceps, leading to white matter damage and pressure injury from retractor blades [15]. The Aurora Surgiscope is a tubular retractor system with circumferential lighting and an integrated visualization system that can be used for minimally invasive approaches to deep-seated, intra-axial lesions while mitigating some of the risks associated with white matter disruption [11]. The port system is also ergonomically beneficial as it eliminates the need for additional tools, such as a robot or microscope, and allows the surgeon to use bimanual techniques with optimal visualization through the port [16]. When the port is toggled or moved, the surgical workflow remains uninterrupted. The system also offers the benefits of even, radial force distribution, minimizing white matter tract damage and reducing the likelihood of ischemic retraction injury, as seen with other port retractors [9].

There are important considerations associated with using the Surgiscope. Intraoperatively, the Surgiscope’s narrow range of working widths and lengths compared to other devices may render the Surgiscope less suitable to treat some lesions [17]. Despite this potential limitation, we were able to use the Surgiscope to resect some larger lesions, including a 6.6-cm glioma. Another challenge can be working with a 2D view projected onto an external monitor at the depth of the port. Neurosurgeons who are accustomed to performing 2D operations using camera and endoscope systems rely on a variety of cues to help overcome the lack of stereoscopic vision, and the same psychomotor skill set applies to the learning curve and use of this camera-port system. Another consideration is that the visualization of the Surgiscope is limited by a small CMOS chip with only 1080P which suffers from artifacts common to short-throw focal length visualization systems. This is unlike the Synaptive exoscope that is often used with the BrainPath system, which can provide 4K imaging [18]. Although the Surgiscope’s short-throw focal system is advantageous for spatial efficiency, reduced glare, and flexibility in placement, this feature may result in optical distortion, limited zoom options, and image quality degradation based on the complex optics required to accommodate its short distance and light brightness limitations [19,20].

Our study is not without limitations. The retrospective, single-institution nature of our study presents inherent limitations. In addition, the limited sample size and heterogeneity within our patient population limits the generalizability of our findings. Clinical trials are ultimately warranted to study the feasibility, efficacy, and safety of the Surgiscope in treating the variety of lesions discussed here. Currently, the EVACUATE trial is evaluating the efficacy of the Surgiscope system in reduction of IPH volume [21], and the MIRROR trial is comparing clinical outcomes between patients who undergo IPH evacuation using the Surgiscope to those who receive standard medical therapy [22]. To date, there have been no randomized control trials that directly compare morbidity and mortality outcomes of deep lesions treated using port-based tubular retractors to conventional open approaches. As a result, the Aurora Surgiscope is an additional, minimally invasive neurosurgical tool that warrants further investigation.

## Conclusions

This is the largest case series to date demonstrating the feasibility of the Aurora Surgiscope used for the resection of deep-seated intracranial lesions. The technology is comparable to other port-based retractor systems with the benefits of integrated visualization and illumination capabilities. Future studies are necessary to evaluate this device in larger patient populations across multiple institutions.
